# Adenine model of chronic renal failure in rats to determine whether MCC950, an NLRP3 inflammasome inhibitor, is a renopreventive

**DOI:** 10.1186/s12882-023-03427-4

**Published:** 2023-12-19

**Authors:** Mahmoud S. Sabra, Fahmy K. Hemida, Essmat A. H. Allam

**Affiliations:** 1 Pharmacology Department, Faculty of Veterinary Medicine, Assiut University, Assiut, 71526 Egypt; 2 Department of Pharmacology and Toxicology, Faculty of Pharmacy, Assiut University, Assiut, 71526 Egypt

**Keywords:** Adenine, CRF, NLRP3, MCC950, Inflammasome, NGAL, Caspase-3, IL-1β

## Abstract

**Background:**

Chronic renal failure (CRF) is defined by a significant decline in renal function that results in decreased salt filtration and inhibition of tubular reabsorption, which ultimately causes volume enlargement. This study evaluated the potential renopreventive effects of the NLRP3 inflammasome inhibitor MCC950 in adenine-induced CRF in rats due to conflicting evidence on the effects of MCC950 on the kidney.

**Methods:**

Since the majority of the kidney tubular abnormalities identified in people with chronic renal disease are comparable to those caused by adding 0.75 percent of adenine powder to a rat's diet each day for four weeks, this method has received broad approval as a model for evaluating kidney damage. Throughout the test, blood pressure was checked weekly and at the beginning. Additionally, oxidative stress factors, urine sample examination, histological modifications, and immunohistochemical adjustments of caspase-3 and interleukin-1 beta (IL-1) levels in renal tissues were carried out.

**Results:**

Results revealed that MCC950, an inhibitor of the NLRP3 inflammasome, had a renopreventive effect, which was demonstrated by a reduction in blood pressure readings and an improvement in urine, serum, and renal tissue indicators that indicate organ damage. This was also demonstrated by the decrease in neutrophil gelatinase-associated lipocalin tubular expression (NGAL).

The NLRP3 inflammasome inhibitor MCC950 was found to significantly alleviate the worsening renal cellular alterations evidenced by increased expression of caspase-3 and IL-1, according to immunohistochemical tests.

**Conclusion:**

The NLRP3 inflammasome inhibitor MCC950 demonstrated renopreventive effects in the CRF rat model, suggesting that it might be used as a treatment strategy to stop the progression of CRF.

## Background

Kidney damage or an estimated glomerular filtration rate less than 60 ml/min per 1.73 square metres are both indicators of chronic renal failure (CRF), which is also known as end-stage renal illness [1]. A progressive decrease of kidney function that eventually necessitates the use of renal replacement therapy is a sign [2]. CRF occurs more frequently, has a bad prognosis, and costs a lot to treat. This illness not only seriously affects people's health but also poses a serious financial challenge for the worldwide health care industry [3]. Rising rates of diabetes and hypertension, which are significant causes of the morbidity and mortality associated with CRF [4, 5], add to the burden of CRF. CRF alone is a significant independent risk factor for the development of cardiovascular illness, including hypertension [6].

Adenine causes chronic renal failure in rats, which manifests as severe renal insufficiency and metabolic abnormalities that are very similar to those seen in uremic people [7]. Additionally, mesangial enlargement, endothelial dysfunction, and glomerular fibrosis are caused by cellular oxidative stress associated with CRF [8].

The NLRP3 inflammasome, which stands for nucleotide-binding and oligomerization domain, leucine-rich repeat, and pyrin domain-containing 3, promotes the development and release of proinflammatory cytokines, which results in excessive inflammatory responses and causes irreparable damage to the body [9]. The NLRP3 inflammasome is implicated in the pathogenesis and progression of chronic renal failure, according to numerous studies [10–13].

A growing body of evidence clearly suggested that the renal tissues of CR have significantly higher levels of NLRP3 expression and caspase-1. For patients with fibrosis, it may be possible to predict that the NLRP3 inflammasome will be involved in the management of renal failure [13–15]. Additionally, research has shown that 5/6-nephrectomized renal models and unilateral ureteral blockage mice both have higher levels of activated NLRP3 inflammasomes in the renal matrix [16, 17]. Adenosine triphosphate hydrolysis to adenosine diphosphate is inhibited by the small molecule MCC950 (Phase II clinical trials), which presents NLRP3 in a closed conformation and prevents oligomerization and activation of the NLRP3 inflammasome [18]. Additionally, it inhibits the processing of cytokines like IL-1 and pyroptosis as a result of NLRP3 activation [19].

Animal illness models for multiple sclerosis [20], traumatic brain injury [21], cryopyrin-associated periodic syndromes [22], Alzheimer's disease, and Parkinson's disease have all shown promise for MCC950 [23, 24]. Additionally, it was demonstrated that MCC950 could reduce diabetic kidney injury by inhibiting the NLRP3/caspase-1/IL-1 pathway [25]. According to a recent study, MCC950 inhibits the NLRP3 inflammasome, which prevents acute renal failure by increasing hypoxia-inducible factor 1 and BCL2/adenovirus E1B interacting protein-mediated mitophagy, which lessens the effects of iohexol-induced apoptosis and renal injury [26]. However, another study reported that the reduction of the NLRP3 inflammasome by MCC950 resulted in a considerable amount of renal inflammation and injury in the model of diabetic kidney mice [27].

Based on the previously mentioned information, efforts have been made to identify effective and precise ways to restrict NLRP3 activation in the group of auto-inflammatory diseases, such as obesity, diabetes, and hypertension. The impact of the inflammasome in human disease studies has prompted these efforts. Therefore, the objective of the current investigation was to evaluate any potential renoprotective benefits of the NLRP3 inflammasome inhibitor MCC950 in rats with CRF caused by adenine.

## Materials and methods

### Animals and chronic renal failure (CRF) induction

The Institutional Animal Care & Use Committee (IACUC) of the Faculty of Medicine, Assiut University, authorized the experimental procedure (approval number: 17300878) and in accordance with ARRIVE guidelines. The study used adult male albino rats (8–10 weeks old) weighing 150–200 g. The animals were kept in the faculty of medicine's animal facility at Assiut University, where they had unrestricted access to food and drink in addition to the typical lighting and darkness patterns of a research lab. Each experimental group contained six to eight animals, chosen at random. Chronic renal failure in rats was induced by adding 0.75 percent weight per weight adenine to the meal every day for four weeks [28].

### Experimental design

Three groups of six to eight rats each were randomly assigned to the animals. In accordance with other investigations, MCC950 sodium (AKSCI, USA) (10 mg/kg/i.p.) was administered to the rats intraperitoneally after being dissolved in phosphate-buffered saline (PBS) [21, 29]. Rats in Group 1 were given PBS as a control throughout a four-week period. Group 2 served as a CRF group and received adenine treatment for four weeks. For four weeks, Group 3 received adenine and MCC950 (10 mg/kg/i.p.) dissolved in saline. During the treatment periods, the rats were weighed weekly. The rats were killed at the end of the studies, and the plasma was collected. Tissues were removed, weighed, and cleaned thoroughly with ice-cold saline. Plasma samples and frozen tissues were kept at − 80 °C.

Rats were euthanized in the following manner: rats were thoroughly anesthetized by inhaling 5% isoflurane. When rats did not respond to stimulation of the head and limbs, they were quickly terminated by cervical dislocation. Rats were declared dead after 10 s of cervical dislocation if they stopped breathing and did not react to systemic stimulation [30].

### Blood pressure measurements

Using a non-invasive tail-cuff technique, blood pressure was monitored before induction and once a week throughout the experiment (Model LE 5001-pressure meter, Panlab, Harvard Apparatus, Spain). To ensure that readings were accurate and corresponded to manufacturer recommendations, rats were trained for three straight days prior to testing.

### Estimation of urinary albumin

Using a readily available commercial kit (Cat. no. DIAG-250- BioAssay Systems-U.S.A.), albumin was assessed in accordance with a set methodology. The intensity of the color detected at 620 nm is directly proportional to the amount of albumin present in the sample [31].

### Estimation of urinary glucose

Utilizing a readily available commercial kit (Cat. no. EGL3-100- BioAssay Systems-U.S. A) and adhering to the recommended procedure, glucose was assessed. The power of the color was determined at 560 nm, which is directly related to the amount of glucose in the sample [32].

### Estimation of urinary ketone bodies

Following the manufacturer's instructions, ketone bodies were assessed using a readily accessible assay kit (Cat. no. EKBD-100-BioAssay Systems-U.S.A.). According to the quantities of acetoacetic acid and 3-hydroxybutyric acid in the sample, the strength of the color was determined at 340 nm [33].

### Assessment of renal functions

Serum creatinine (Cat. no. 234–000) and blood urea nitrogen (Cat. no. UR 21–10) were measured for renal functions using commercially available kits (Schiffgraben, Hannover, Germany) in accordance with the prescribed methodology. A spectrophotometer is used to measure the aforementioned parameters.

### Markers of the oxidative stress

According to an earlier study (Janero, 1990), malondialdehyde was assessed spectrophotometrically in kidney tissue samples using commercially available kits (Schiffgraben, Hannover, Germany) (Cat. no. MD 25–28), nitric oxide (NO) contents (Cat. no. NO 25–33) as previously stated [34], and reduced glutathione (GSH) (Cat. no. GR 25–11) as [35].

### Selective biomarker NGAL for assessing chronic renal failure

After damage, the rat kidneys exhibit high levels of neutrophil gelatinase-associated lipocalin (NGAL), a member of the lipocalin superfamily, particularly at the proximal convoluted tubule. A commercial ELISA kit (Cat. no. E-EL-R0662, Sunlong, Biotechnology, Hangzhou, Zhejiang, China) was used to measure NGAL [36].

### Histopathological and immunohistochemical studies

For a 24-h period, kidney tissue was fixed with 10% formalin before being dehydrated and embedded in paraffin. Hematoxylin–eosin staining was applied to sections of kidney tissue that were approximately 4 microns thick (H&E). In each segment, 20 randomly chosen locations were subjected to blinded inspection for light microscopic investigation [37].

The immunohistochemistry analysis of caspase-3 and interleukin-1 beta (IL-1β) was performed. Paraffin was removed from tissue Sects. (4 m thick) in xylene before they were rehydrated. The slices were immersed in 3 percent H2O2 for 10 min to remove endogenous peroxidase activity and then rinsed with PBS (2 min X 3 times). The slices were then incubated with primary polyclonal rabbit active anti-caspase-3 antibody in dilution 1/200 (E-AB-6602, Elabscience Biotechnology, Inc, USA) and polyclonal rabbit anti-IL-1 antibody; 1/100 (E-AB-66749, Elabscience Biotechnology, Inc, USA) for 1 h at room temperature. This was done in accordance with the standard manufacturing protocol (Vector Laboratories, Burlingame, CA) Then, for 20 min, polyperoxidase-anti-Mouse/Rabbit IgG was added. A streptavidin–biotin-peroxidase kit was used to detect the antigen–antibody complex, and Mayer's hematoxylin was used as a counterstain. For each experiment, there were positive and negative control sections.

Intense brown nuclear and cytoplasmic staining allowed the active caspase-3 and IL-1beta immunostaining cells to be distinguished. The number of immunopositive cells in each slide was counted in 5 distinct microscopic fields, and the mean number for each slide was computed, followed by the mean ± SE for each group. In the 20 randomly chosen regions, the sections were examined using an Olympus BX41 light microscope at a high magnification (X400). We used a digital camera to take photomicrographs (ToupCam LCMOS05100KPA).

### Statistical analysis

Data for each evaluated parameter were tested for the normality of distributions (Shapiro–Wilk test, *p* > 0.05). Statistical analysis was assessed by one-way ANOVA followed by Tukey’s post hoc test. For repeated measures (blood pressure), data were analyzed with two-way ANOVA with repeated measures followed by the Bonferroni post hoc test. Scatter dot plots and arithmetic mean ± standard error of the mean (SEM) are used to display data. Data of *p* ≤ 0.05 was considered statistically significant. Graph Pad prism® software (version 8) was used to perform these statistical investigations.

## Results

### Normality of distributions

Comparing the Shapiro–Wilk test against other tests including the Kolmogorov–Smirnov, Lilliefors, and Anderson–Darling tests, it was shown to be the most effective at determining whether data had normal distributions. The Shapiro–Wilk test significance values for the investigated parameters were higher than 0.05. Therefore, the current study's data were normal.

### The effect of MCC950 on body weights and relative kidney weight in rats with adenine-induced CRF

**Fig. 1:**
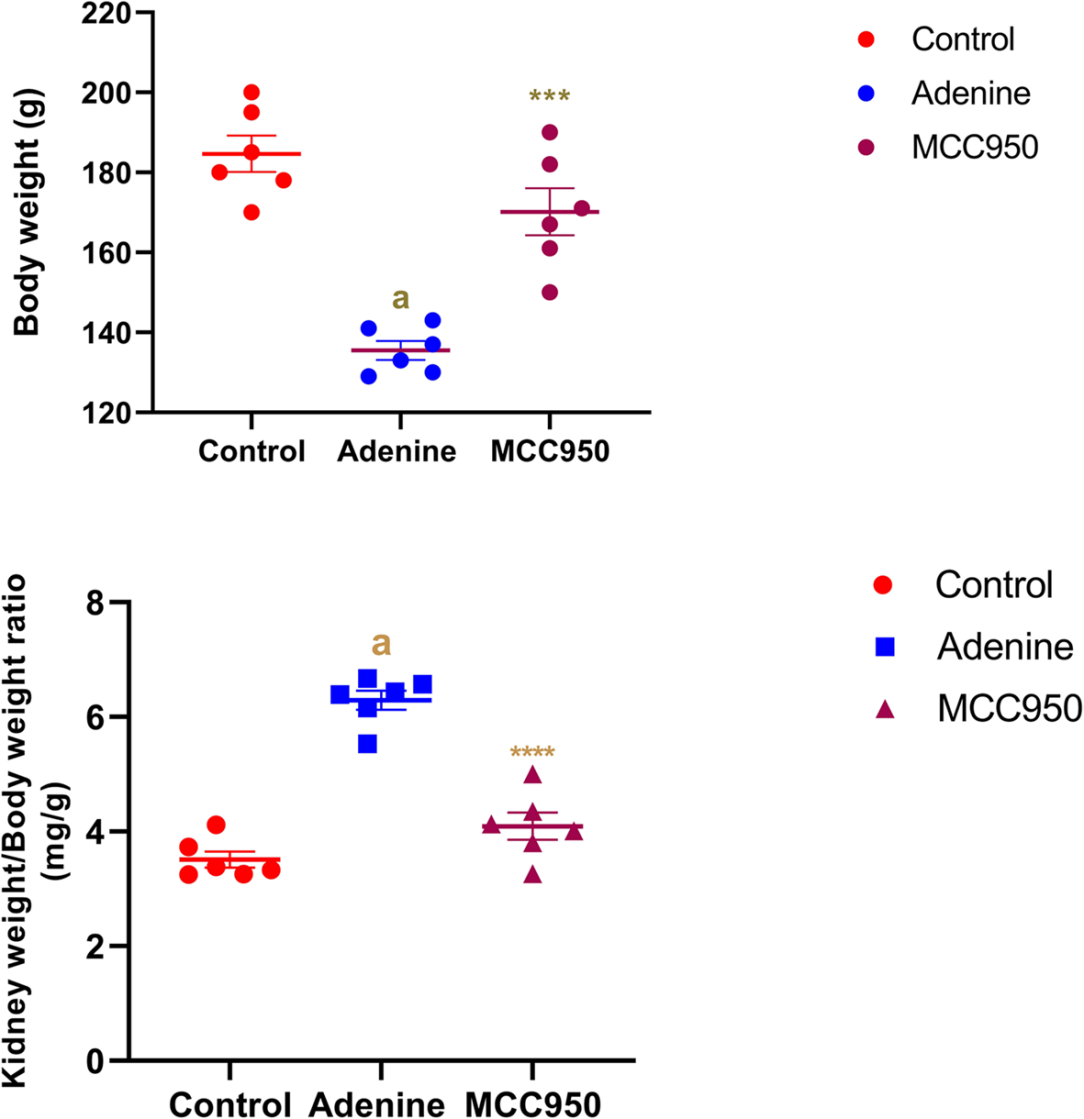
Effect of MCC950 on body weight and relative kidney weight on adenine-induced chronic renal failure in rats. Data are the means ± SEM (*n* = 6). ***p < 0. 001 and ****p < 0.0001 is comparable to the adenine-treated group. **a** *P* < 0.0001 in comparison to the control group

A drop in body weight was found in CRF rats, which was changed by MCC950 (Fig. 1). Furthermore, as compared to the control group, CRF rats had increased relative kidney weight, which were substantially reduced by MCC950 therapy (Fig. 1).

### Effect of the MCC950 on arterial blood pressure in adenine induced CRF in rats

**Fig. 2:**
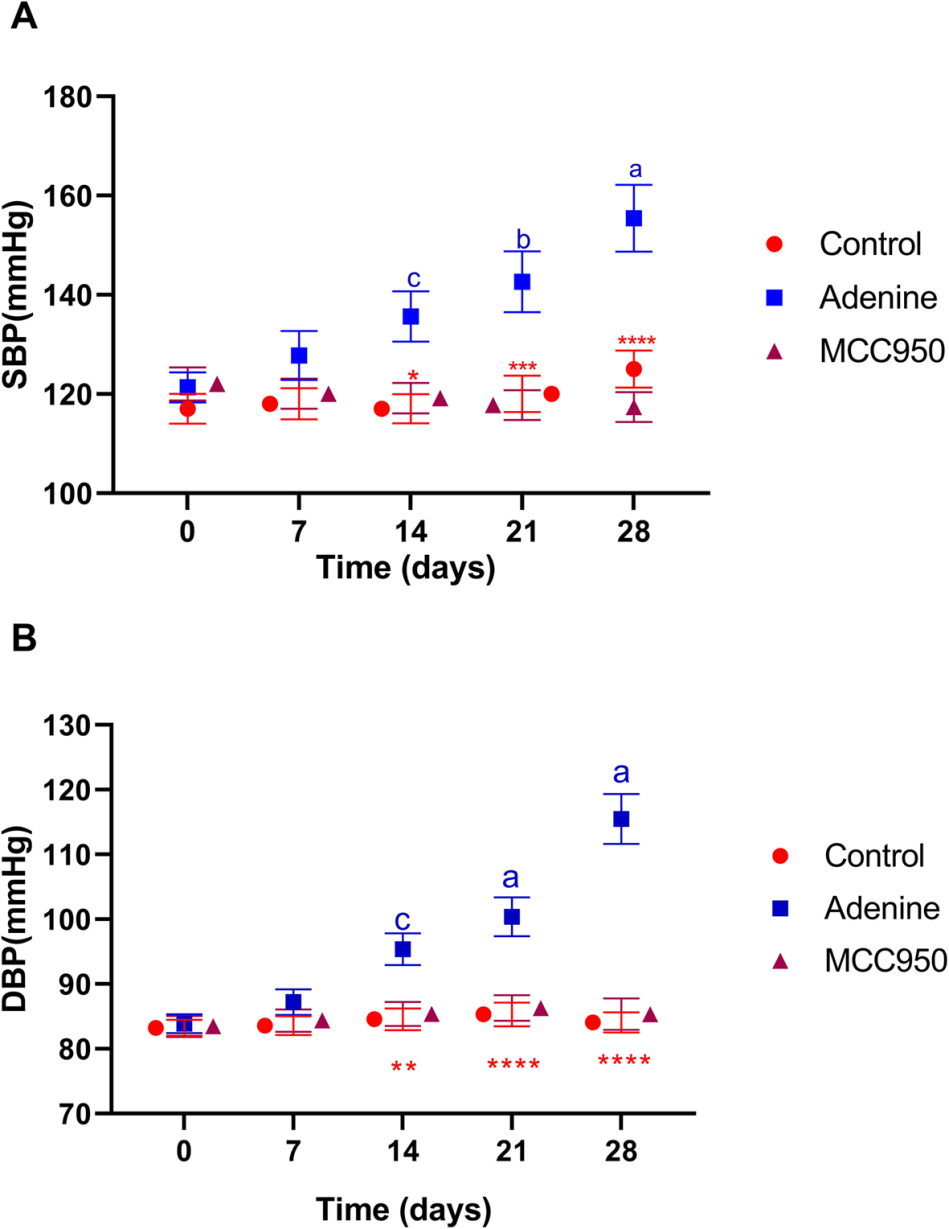
Effect of MCC950 on systolic (1A) and diastolic (1B) blood pressure on adenine-induced chronic renal failure in rats. Data are the means ± SEM (*n* = 6). ****p < 0.0001, ***p < 0. 001, **p < 0.01 and *p < 0.05 as compared to the adenine group. **a** *P* < 0.0001 in comparison to the control group. **b** *P* < 0.001 in comparison to the control group. **c** *P* < 0.01 in comparison to the control group

As seen in Fig. 2 (A, B), rats treated with adenine had a substantial and significant (*p* < 0.0001 and *p* < 0.0001) rise in both systolic and diastolic blood pressure compared to the control group. After the course of treatment, the systolic blood pressure increased from 125.034 ± 3.0710 mmHg in the control group to 155.430 ± 6.754 mmHg in the group that received adenine. Diastolic blood pressure levels in animals that were persistently given adenine increased from 84.08 ± 1.56 mmHg in control rats to 115.49 ± 3.84 mmHg in those animals. Adenine-induced blood pressure increase is significantly (*p* < 0.0001 and *p* < 0.0001) prevented by concurrent administration of MCC950.

### Effect of MCC950 on urinary levels of albumin, glucose and ketone bodies in adenine-induced CRF in rats

**Fig. 3:**
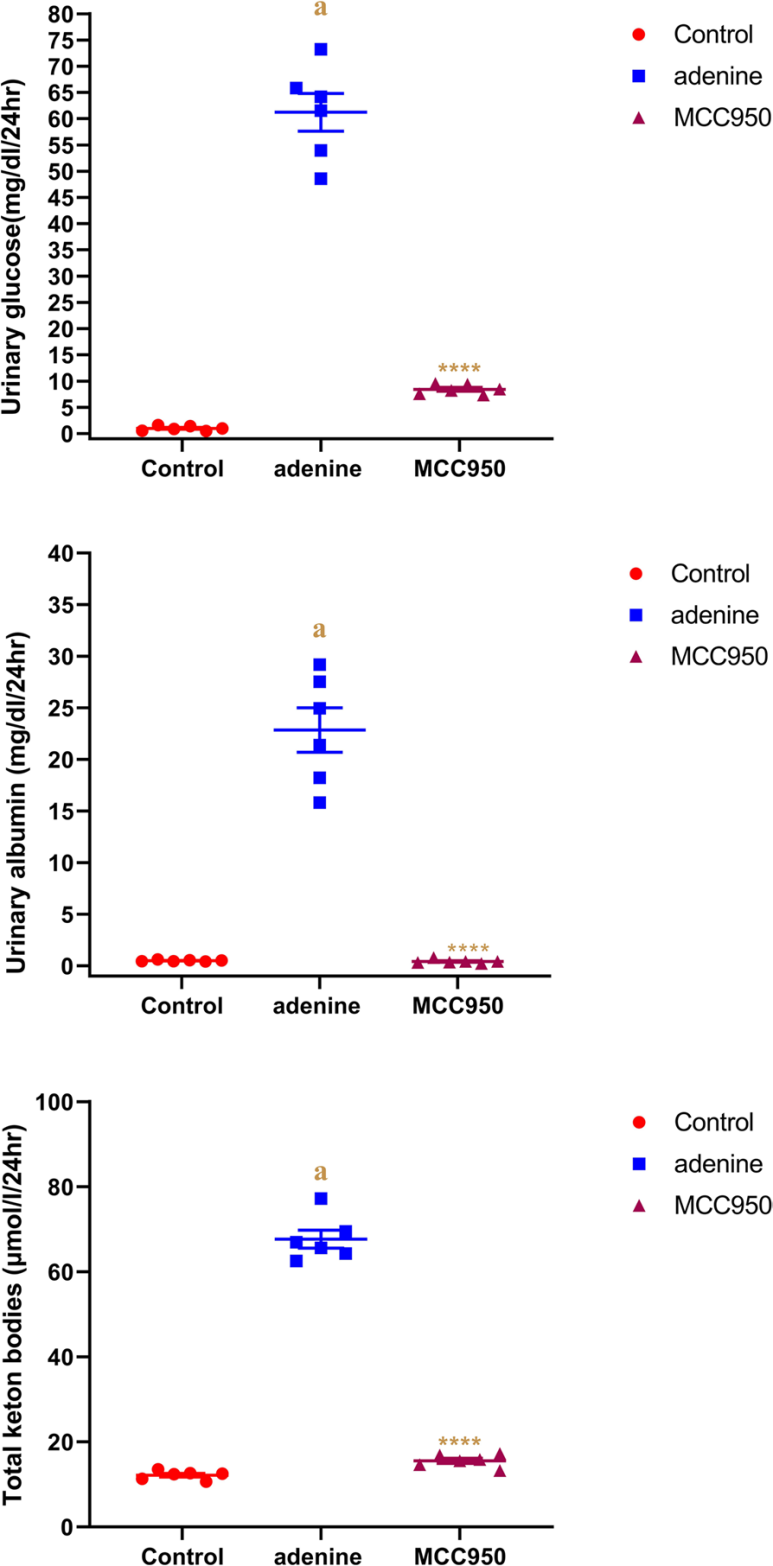
Effect of MCC950 on glucose, albumin, and total ketone bodies levels in rat’s urine in adenine-induced chronic renal failure. Data are the means ± SEM (*n* = 6). ****p < 0.0001 is comparable to the adenine-treated group. **a** *P* < 0.0001 in comparison to the control group

Rats with adenine-induced CRF had significantly higher urine levels of albumin, glucose, and ketone bodies than those in the PBS control group (p 0.0001). Figure 3 illustrates how MCC950 showed a significant reduction (*p* < 0.0001) in urine levels of albumin, glucose, and ketone bodies compared to CRF-induced animals.

### Effect of MCC950 on serum creatinine level and urea level in adenine-induced CRF in rats

**Fig. 4:**
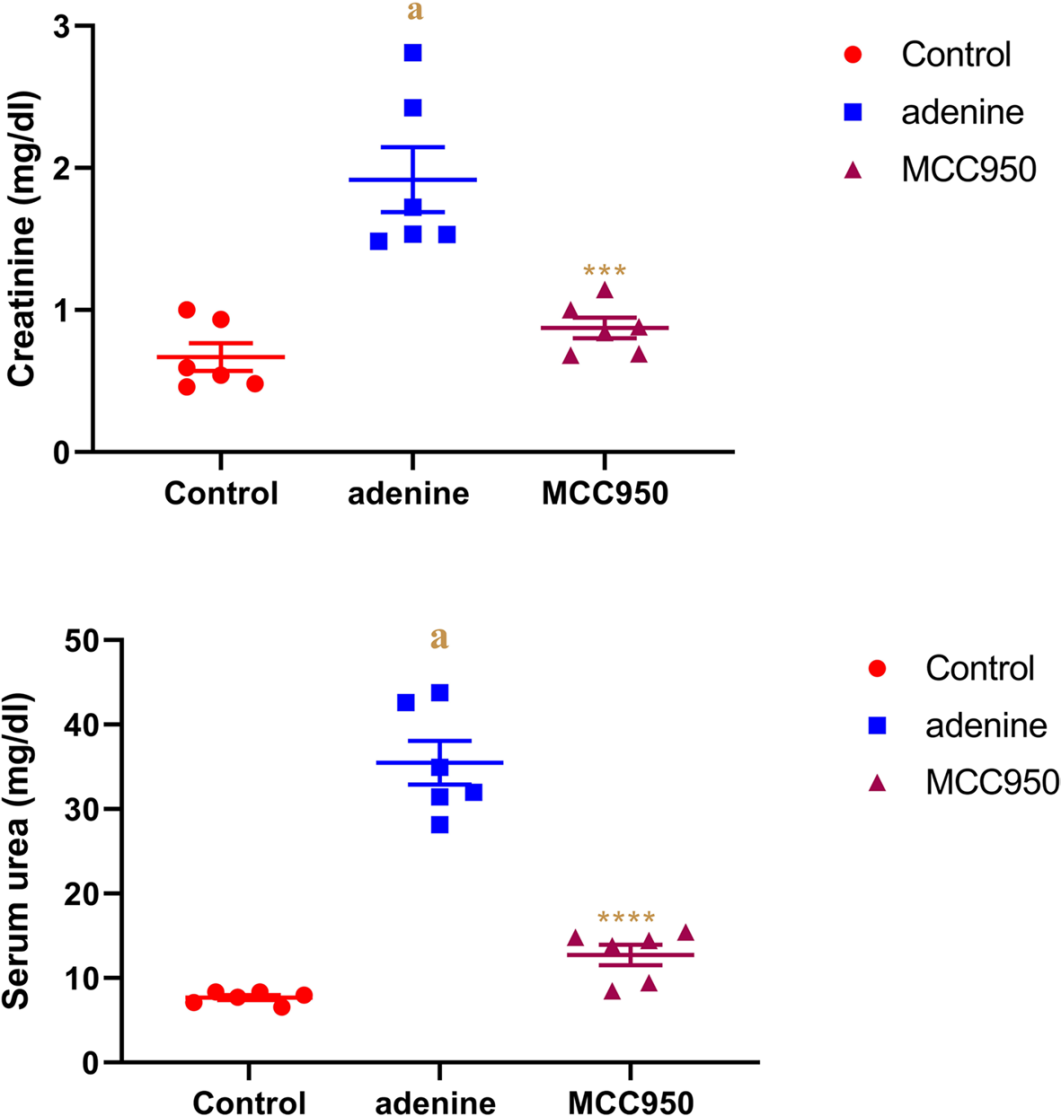
Effect of MCC950 on creatinine and urea levels in rat’s serum in adenine-induced chronic renal failure. Data are the means ± SEM (*n* = 6). ****p < 0.0001 and ***p < 0. 001 are comparable to the adenine group. **a** *P* < 0.0001 in comparison to the control group

Rats with adenine-induced CRF had significantly higher blood concentrations of urea and creatinine (*p* < 0.0001) compared to the PBS control group. As indicated in Fig. 4, MCC950 demonstrated a significant decline (*p* < 0.001) in these levels as comparable to CRF-induced animals.

### Effect of MCC950 on tissue malondialdehyde, reduced glutathione and nitrite levels in adenine-induced CRF in rats 

**Fig. 5:**
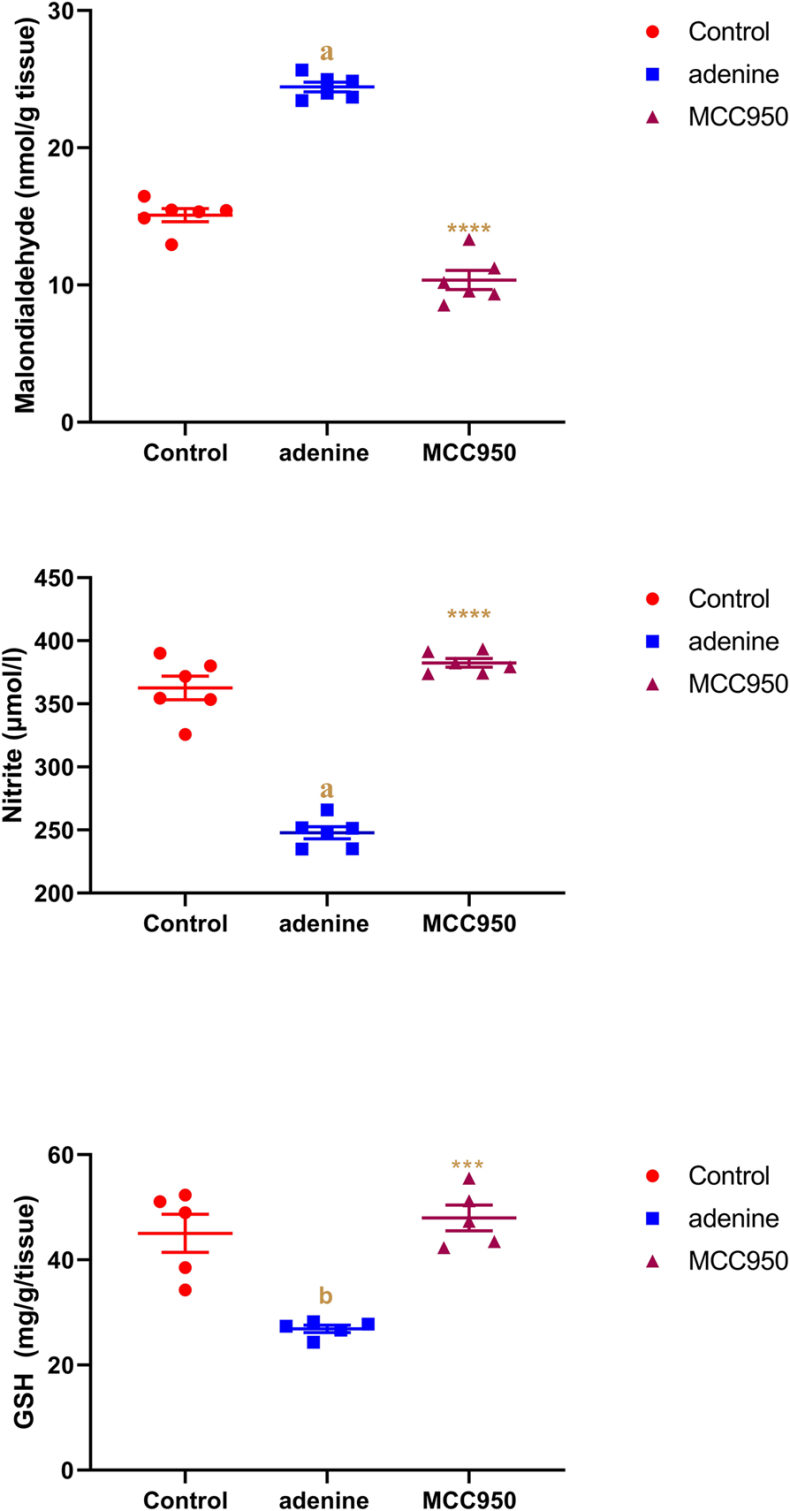
Effect of MCC950 on malondialdehyde, nitrite, and reduced glutathione (GSH) levels in rat’s kidney tissue in adenine-induced chronic renal failure. Data are the means ± SEM (*n* = 6). ****p < 0.0001 and ***p < 0. 001 as compared to the adenine group. **a** *P* < 0.0001 in comparison to the control group. **b** *P* < 0.001 in comparison to the control group

As presented in Fig. 5, CRF-induced rats showed a significant increase (*p* < 0.0001) in tissue malondialdehyde and a significant reduction in nitrite (*p* < 0.0001) and reduced glutathione (*p* < 0.001) levels compared to the PBS control group. Treatment by MCC950 exhibited a significant decrease (*p* < 0.0001) in tissue malondialdehyde while an increase in nitrite (*p* < 0.0001) and glutathione (*p* < 0.001) levels compared to CRF-induced rats.

### Effect of MCC950 on tissue neutrophil gelatinase-associated lipocalin (NGAL) levels in adenine-induced CRF in rats

**Fig. 6:**
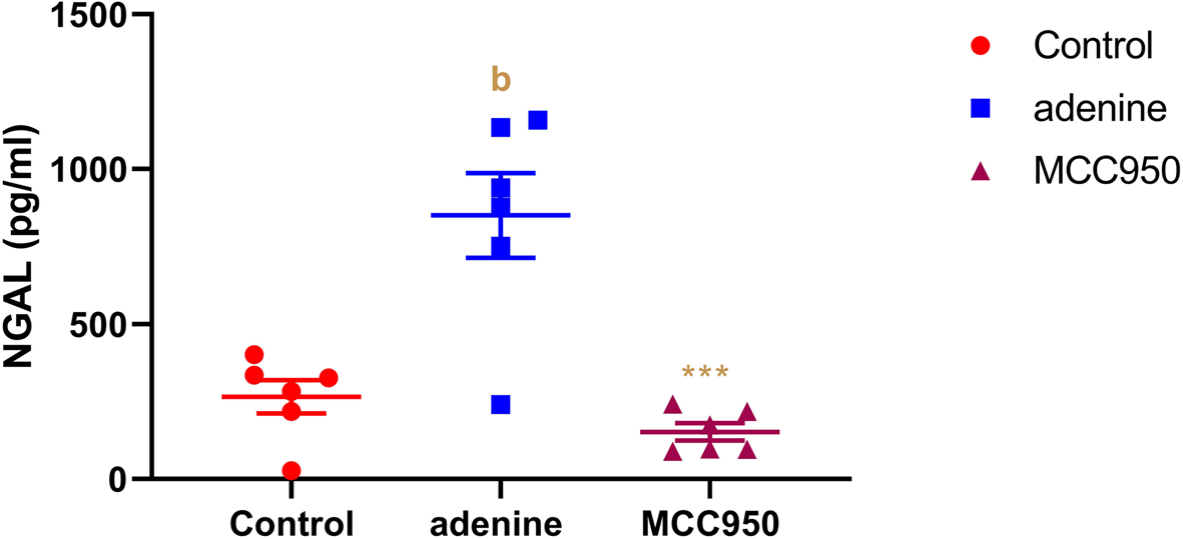
Effect of MCC950 on neutrophil gelatinase-associated lipocalin (NGAL) levels in rat’s kidney in adenine-induced chronic renal failure. Data are the means ± SEM (*n* = 6). ***p < 0.001 in comparison to the adenine group. **b** *P* < 0.001 in comparison to the control group

As shown in Fig. 6, when compared to the PBS control group, the tissue NGAL of the adenine-induced CRF rats increased noticeably (*p* < 0.001). Comparing the MCC950-treated group to the CRF-induced group, tissue NGAL showed a substantial reduction (*p* < 0.001).

### Histopathological alterations in CRF-induced rat model

**Fig. 7:**
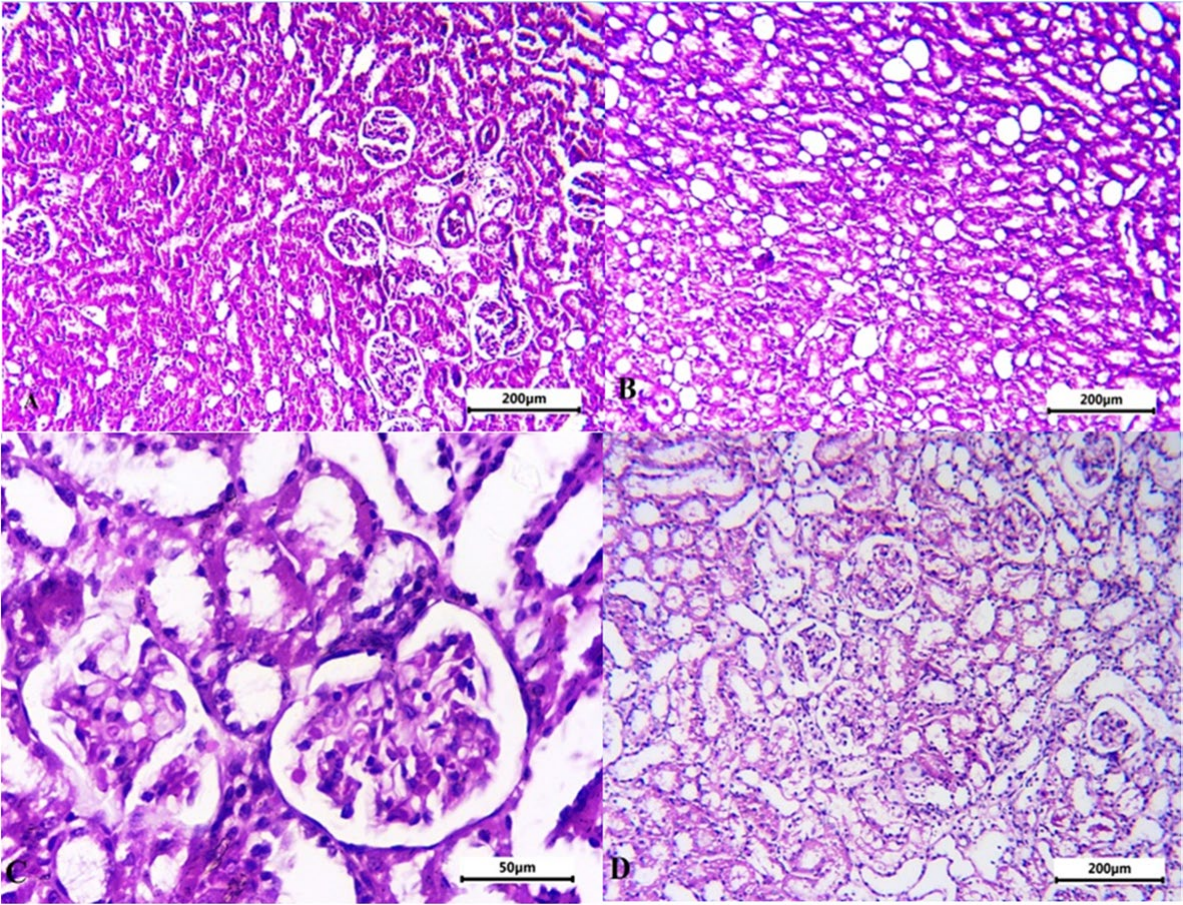
Representative micrograph of the kidney of the studied group stained with H&E. **A** Control negative group showing normal architecture of the Kidney. **B**, **C** Adenine induced CRF group showing vacuolar degeneration of the renal tubule, focal area of mononuclear cell infiltration and loss of architecture of the glomeruli with destruction of the Bowman's capsule. **D** MCC950 treated group showed improvement of the tubular cells with recovery of the architecture of the glomeruli

The histopathological lesion score of all groups tested was summarized in (Table 1). The kidneys in the control negative group were found to have normal architecture. Tubular and glomerular alterations were seen in the adenine-induced CRF group. The tubular abnormalities were vacuolar degeneration with mononuclear cell infiltration, whereas the glomerular changes were glomerulolysis with loss of architecture and disintegration of Bowman's capsule. The tubular cells in the MCC950-treated group improved, and the morphology of the glomeruli recovered (Fig. 7).

**Table 1:** Summary of the lesion score of the studied groups

**Group Lesion**	Control -ve	Adenine-induced CRF group	MCC950 treated group
Vacuolar degeneration	-	+ + +	+
Mononuclear cell infiltration	-	+ + +	+ +
Loss of glomeruli architecture	-	+ + +	-
Destruction of Bowman's capsule	-	+ + +	-

- No lesions, + lesions present in 2–3 sections, +  + lesions present in 4–7 sections, +  +  + lesions present in 8–10 sections

### Immunohistochemistry alterations in caspase-3 and interleukin- 1 beta (IL-1β) expression in CRF-induced animals

**Fig. 8:**
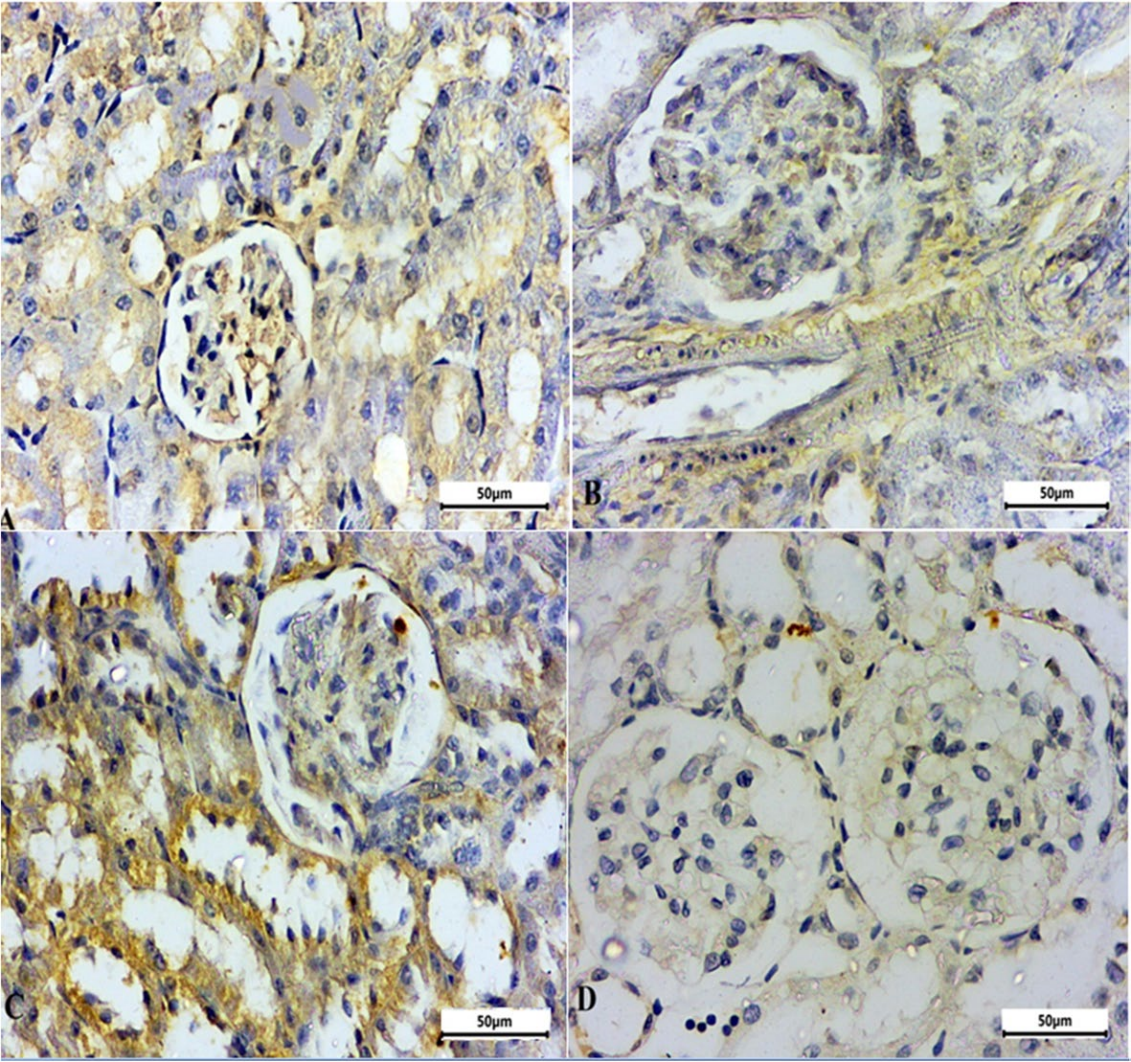
Representative micrograph of the kidney of the studied group stained with IL-1beta. **A** Control negative group, **B**-**C** adenine induced CRF group and **D** MCC950 treated group in adenine rat model

**Fig. 9:**
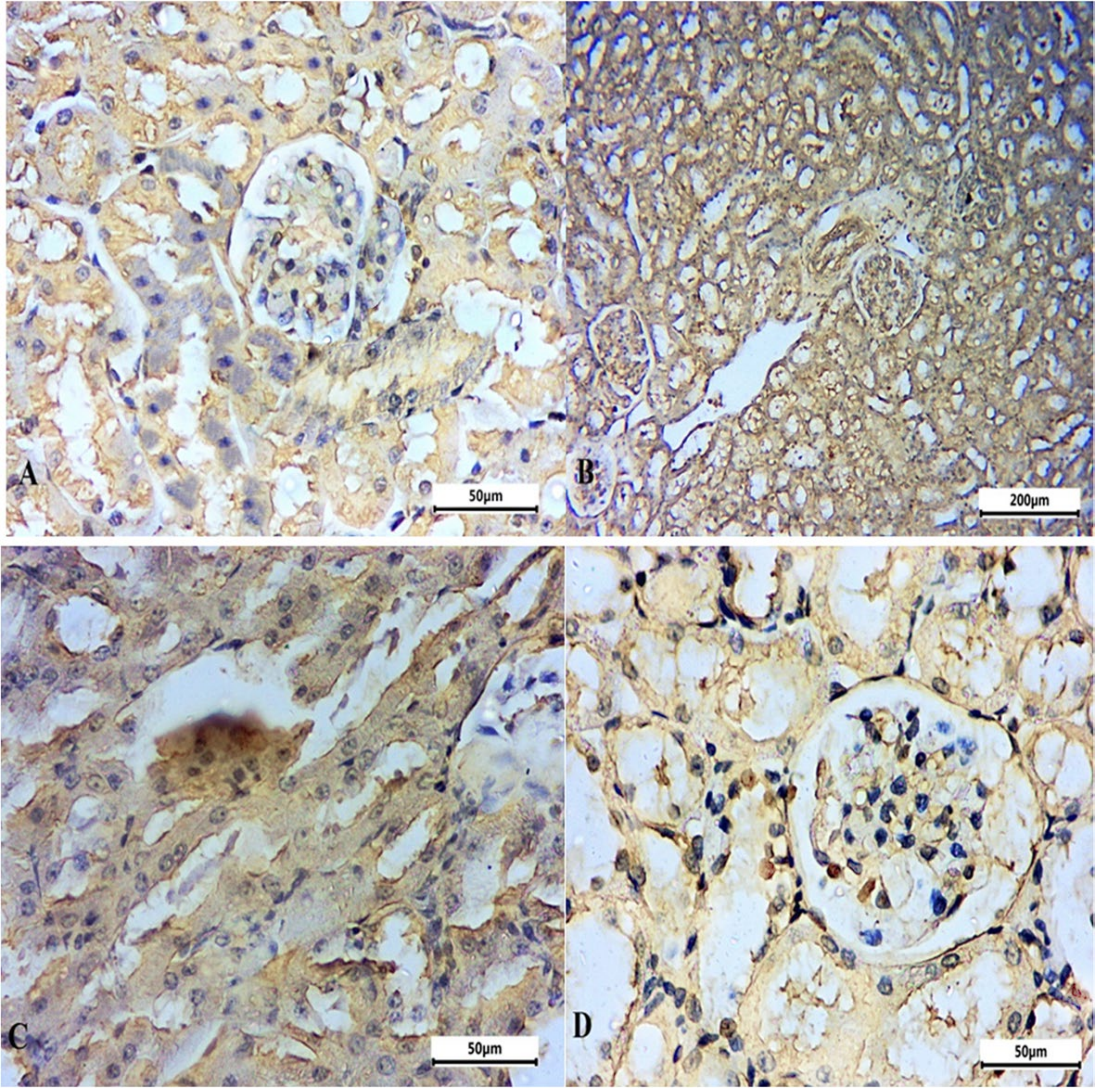
Representative micrograph of the kidney of the studied group stained with Caspase-3. **A** Control negative group, **B**-**C** adenine induced CRF group and **D** MCC950 treated group in adenine rat model

**Fig. 10:**
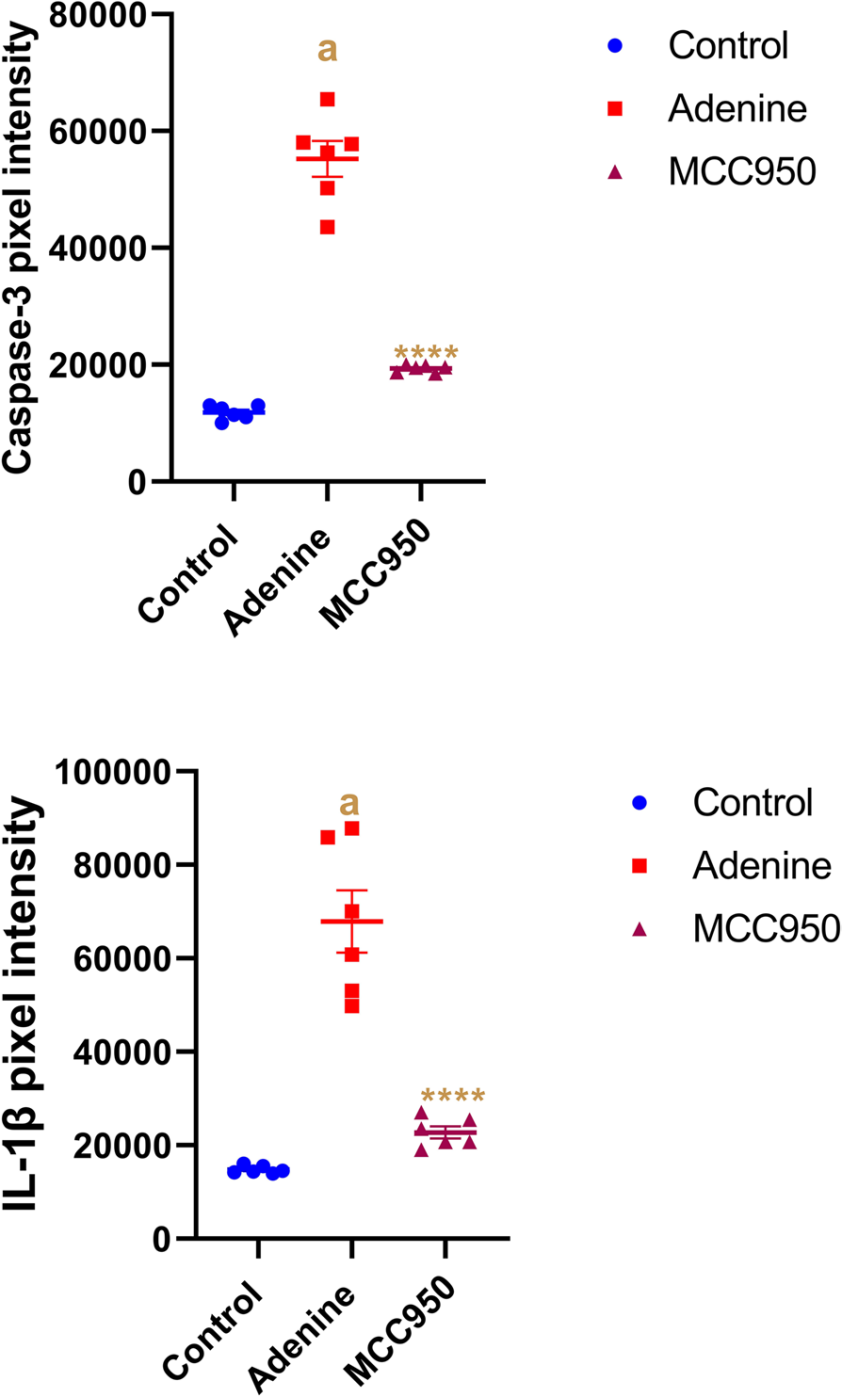
Quantitative immunohistochemistry analysis. Integrated pixel intensity analysis of caspase-3 and interleukin-1β (IL-1β). Data are the means ± SEM. ****p < 0.0001 as compared to the adenine group. **a** *P* < 0.0001 in comparison to the control group

Immunohistochemical staining of IL-1β and caspase-3 in rat kidney (Table 2, Figs. 8, 9 and 10) shows the immunoreactivity results for IL-1β and caspase-3 respectively. IL-1β (Fig. 8) and caspase-3 (Fig. 9) activity was moderately low in control group tubular epithelial cells (Figs. 8A, and 9A). IL-1β immunoreactivity and caspase-3 immunoreactivity were significantly increased in adenine CRF-induced rats (Fig. 8B-C and Fig. 9B-C). In MCC950 treated group there was a significant reduction in immunoreactivity for IL-1β and caspase-3 (Figs. 8D and 9D).

**Table 2:** Effects of MCC950 treatment in adenine-induced chronic renal failure (CRF) on the percentage expression of interleukin-1 beta (IL-1β) and caspase-3 in the kidney of rats

**Group Immune stain**	Control -ve	Adenine-induced CRF group	MCC950 treated group
IL-1beta	14.52^a^	49.75^b^	22.74^a^
Caspase-3	11.05^a^	44.06^b^	18.50^a^

^a,^^b^ values are not sharing a common superscript letter differ significantly at *P* < 0.05

## Discussion

A vital prerequisite for improving the prognosis for CRF patients is an efficient treatment or prevention [38]. The innate immune system's primary components, the inflammasomes, respond to cellular stress by boosting the production of pro-inflammatory cytokines like IL-1 and IL-18 and by promoting pyroptosis, an inflammatory form of cell death [39]. A number of renal diseases, including acute renal failure [40], chronic renal failure [41], diabetic nephropathy [42], and crystal-related nephropathy have been linked to the NLRP3 inflammasome [43].

The initial adenine diet paradigm resulted in renal disease with early onset, substantial tubulointerstitial fibrosis, tubular atrophy, crystal formation, and significant vessel calcification. Lower adenine consumption in rats has now been demonstrated to cause gradually escalating renal impairment and cardiovascular disease. These chronic adenine diet models enable the study of relatively constant renal and cardiovascular disease, which is similar to CRF in people. Cardiovascular disease is a major source of increased morbidity and death in CRF patients, however the relationships between CRF and cardiovascular disease are poorly understood [44]. Renal vascular disease has been defined as a disorder in which the primary renal arteries gradually occlude, causing hypertension and renal hypoperfusion, eventually leading to chronic renal ischemia [45]. Adenine could elevated serum urea nitrate, serum creatinine concentrations, and uric acid excretion in urine in this model, caused proteinuria, and promoted renal tubulointerstitial nephritis and fibrosis, which mimicked CRF in people [46].

Furthermore, the model resulted in a significant decrease in free amino acids and calcium concentration, hypoalbuminemia, increased albuminuria, hyperlipidemia, and vascular calcification, all of which are features found in clinical CRF. The kidneys were significantly enlarged, with interstitial inflammatory infiltrates and crystalline tubulointerstitial deposits, as well as oxidative stress, resembling a human CRF mechanistic route [47]. Also, adenine caused rapid onset kidney damage, increasing blood urea nitrogen and serum creatinine concentrations within 6 days, causing apoptotic lesions in 70–80 percent of kidney tissue within 4 weeks, and causing distended fibrotic kidneys with a granulated attendance [44].

In the current investigation, therapy with the NLRP3 inflammasome inhibitor MCC950 significantly decreased blood pressure, albumin, glucose, and ketone body levels in the urine, as well as serum levels of urea and creatinine in rats with adenine-induced CRF.

Instead of end-stage renal disease, hypertension is one of the main causes of mortality and morbidity in people with CRF [48]. Thus, the impact of MCC950 on blood pressure was assessed in this study. The findings demonstrated that long-term adenine feeding was linked to higher systolic and diastolic blood pressure. Long-term adenine therapy increases blood pressure, according to studies by [7, 49]. Animals received both adenine and the MCC950; the latter successfully prevented the rise in blood pressure.

Previous study looked into the efficacy of MCC950 in lowering blood pressure and preventing renal fibrosis, inflammation, and dysfunction in mice with pre-existing hypertension [50]. In that study, hypertensive C57BL6/J mice were treated for 11 days with three different doses of MCC950 (2, 5, and 10 mg/kg/day) to determine the best antihypertensive dosage, which was 10 mg/kg/day. Also, Zhang and colleagues investigated the effectiveness of MCC950 on mouse models of type 2 diabetes at a dosage of 20mg/kg and discovered that MCC950 might be used as a tool for primary prevention of diabetic kidney damage [25]. On the other hand, another study found that MCC950's suppression of the NLRP3 inflammasome led to severe renal inflammation and injury in a model of diabetic kidney mice. In the study of Østergaard and colleagues, MCC950 was administered at a total weekly dose of 15 mg/kg i.p. in non-diabetic and diabetic apolipoprotein E knockout mice with established diabetic kidney disease, resulting in increased renal injury and macrophage infiltration in association with increased cellular oxidative stress, as well as increased mesangial expansion and glomerulosclerosis via upregulation of inflammatory and fibrotic markers [27]. A plausible reason for these disparate results might be found in the multiple mice models of diabetes studied, which differed from our model, as well as the distinct MCC950 dosing regimen employed.

Due to glomerular and tubular damage brought on by CRF, both protein filtration and reabsorption mechanisms become dysfunctional, resulting in increased levels of albumin and glucose excretion in the urine [51, 52]. Additionally, the kidney cortex and medulla shared some degree of sustained elevation in free fatty acids and ketone bodies as a result of chronic renal ischemia [53, 54]. Given that the serum levels of creatinine are influenced by glomerular filtration rate, the rise in creatinine and blood urea nitrogen is regarded as a significant indicator of renal damage [55]. Furthermore, in the adenine model of CRF, proteinuria might be produced by injury to the glomeruli or renal tubules caused by the precipitation of 2,8-dihydroxyadenine, an adenine metabolite.

When serum creatinine levels double over the usual amount due to renal damage, the ability to filter out creatinine is reduced, increasing creatinine levels, and the glomerular filtration rate is thought to have been reduced by half [56]. Increased protein catabolism or the conversion of ammonia to urea as a result of increased enzyme production involved in urea assembly are both associated with increased blood urea nitrogen [57]. In the same way, a study reported a reduction in urinary albumin to creatinine ratio and decreased serum creatinine related to kidney injury improvement by MCC950 treatment in a mice model of diabetic nephropathy [25]. Another study demonstrated that ozone therapy's modulation of the NLRP3 inflammasome in the 5/6 nephrectomized CRF rat model led to an improvement in renal function and a decrease in serum urea and creatinine [41].

Reactive oxygen species (ROS) and NLRP3 inflammasome interactions help control immunological reactions during inflammation. According to studies, the activation of the NLRP3 inflammasome has a minor influence on ROS generation [58, 59]. Additionally, ROS may cause the NLRP3 components that are involved in inducing apoptosis in innate immune cells during inflammation [60]. When renal proximal tubular epithelial cells are damaged, it is seen that the expression of oxidative stress markers increases and the activity of the antioxidant enzyme superoxide dismutase decreases. This results in the activation of the NLRP3 inflammasome and proinflammatory pathways, including nuclear factor-kB [61, 62].

The pathophysiology of CRF is significantly influenced by reactive oxygen species. Free oxygen radicals can cause the cell membrane's lipid to oxidize, which can result in renal tubular cell necrosis [63]. Furthermore, it is well-known that intracellular reduced glutathione, the most effective non-enzymatic antioxidant involved in neutralizing free radicals, regulates the cellular redox environment to control a variety of cellular processes, including gene expression, cell-cycle progression, apoptosis, and metabolism [64]. Nitric oxide deficit that is less severe than decreased kidney nitric oxide production and/or increased nitric oxide bio-inactivation may have a role in the development of CRF [65].

In the present work, we discovered that treatment with MCC950 significantly reduced levels of oxidative stress indicators such malondialdehyde, significantly increased levels of the antioxidant enzyme GSH, and significantly increased levels of nitric oxide in the adenine-induced CRF rat model. These findings are consistent with earlier study which suggested that NLRP3 suppression with MCC950 therapy may prevent the development of chronic kidney disease brought on by cisplatin by reducing oxidative stress and inflammation [62].

The kidneys, trachea, and digestive system all have low levels of NGAL expression when subjected to typical physiological conditions. On the other hand, when there is damage, NGAL secretion increases quickly in the thick ascending limb of the renal tubules [66]. In addition, NGAL closely reflects the pattern of renal impairment in CRF patients and is a powerful and independent risk factor for CRF development [67]. A recent study investigated that NGAL has the potential to be an appropriate biomarker for CRF identification in the early stages [68]. In this investigation, we found that NGAL, which has long been recognized as a sign of CRF, was significantly elevated in rats with adenine-induced CRF. These findings support the findings of [69] study, which found that NGAL was elevated in adenine-induced CRF. Additionally, it showed that NGAL participates in nephrogenic regeneration and healing during the course of kidney injury [70]. The current study further shown that prolonged therapy with MCC950 for 28 days dramatically reduced tissue NGAL levels in adenine-induced CRF to levels that were nearly identical to the PBS group. By blocking the NLRP3/caspase-1/IL-1 pathway and lowering urinary NGAL levels in mice with a model of diabetic nephropathy, researchers demonstrated that MCC950 might significantly alleviate diabetic chronic kidney injury [25]. Additionally, in the remnant kidney rat model of CRF, suppression of the NLRP3 inflammasome lowers the levels of NGAL in the urine [71].

Microscopical analysis in the adenine rat model demonstrated tubulointerstitial damage with infiltrating leukocytes, interstitial edoema, and widening of the bowman's space, according to previous histological findings in CRF [72]. These conclusions agree with those of the current investigation on the histological changes in adenine-induced CRF. The larger improvement was evident in the current investigation when MCC950 was administered to adenine-induced rats for 28 days, and this improvement was indicated by histological improvement in renal tissues.

Through inflammatory cytokines, CRF formation and progression are controlled. Additionally, it is well acknowledged that the levels of numerous inflammatory cytokines, including IL-6, IL-1, and tumor necrosis factor-, were higher in CRF patients [73]. Adenine administration may increase the plasma levels of several inflammatory cytokines, including tumor necrosis factor and IL-1 [69]. The pathophysiology of CRF is strongly influenced by oxidative stress, which can also lead to mitochondrial apoptosis and worsen renal failure [74]. The apoptotic protein caspase-3 and the inflammatory mediator IL-1 were therefore examined. Moreover, research suggested that adenine-induced CRF may include caspase-3 [75].

An earlier investigation revealed that the activation of caspase-3 and -7 resulted in the activation of the NLRP3 inflammasome and the oversecretion of IL-1 [76]. Additionally, the NLRP3/caspase-3 pathway's effects on the cell cycle and apoptosis may contribute to the formation of myeloid cells [76]. Through a considerable decrease in the protein expression of both interleukin 1 and caspase-3, the present study demonstrated a significant improvement in the adenine-induced CRF rats treated with MCC950. Not in line with the findings of [25], who demonstrated that blocking the NLRP3/caspase-1/IL-1 pathway with MCC950 efficiently repaired diabetic kidney damage. In general, the current data strongly indicate the renoprotective action of MCC950 in adenine-induced CRF. These findings are confirmed by the biochemical, histological, and immunohistochemistry outcomes of this investigation.

## Conclusion

The treatment of the NLRP3 inflammasome inhibitor MCC950 in the CRF rat model demonstrated renopreventive effects, raising the possibility of a therapeutic strategy to stop the progression of CRF.

### Limitation section

The study has some limitations that should be acknowledged. Firstly, the adenine-induced chronic renal failure model used may not accurately represent all facets of human kidney disease. Secondly, the study did not assess molecular changes in kidney tissue, incorporating analyses such as Western blotting and gene expression could potentially clarify the mechanisms underlying the observed morphological improvements with MCC950 treatment. Lastly, the study did not measure additional parameters like blood glucose, HbA1c, and plasma cholesterol. Including these metabolic markers in future studies could offer further insight into the effects of MCC950 on the progression of renal failure.

## Data Availability

The datasets generated during and/or analyzed during the current study are available from the corresponding author on reasonable request.
